# Signed random walk diffusion for effective representation learning in signed graphs

**DOI:** 10.1371/journal.pone.0265001

**Published:** 2022-03-17

**Authors:** Jinhong Jung, Jaemin Yoo, U. Kang

**Affiliations:** 1 Seoul National University, Seoul, Republic of Korea; 2 Jeonbuk National University, Jeonju, Republic of Korea; Valahia University of Targoviste: Universitatea Valahia din Targoviste, ROMANIA

## Abstract

How can we model node representations to accurately infer the signs of missing edges in a signed social graph? Signed social graphs have attracted considerable attention to model trust relationships between people. Various representation learning methods such as network embedding and graph convolutional network (GCN) have been proposed to analyze signed graphs. However, existing network embedding models are not end-to-end for a specific task, and GCN-based models exhibit a performance degradation issue when their depth increases. In this paper, we propose Signed Diffusion Network (SidNet), a novel graph neural network that achieves end-to-end node representation learning for link sign prediction in signed social graphs. We propose a new random walk based feature aggregation, which is specially designed for signed graphs, so that SidNet effectively diffuses hidden node features and uses more information from neighboring nodes. Through extensive experiments, we show that SidNet significantly outperforms state-of-the-art models in terms of link sign prediction accuracy.

## Introduction

Given a signed social graph, how can we learn appropriate node representations to infer the signs of missing edges? Signed social graphs model trust relationships between people with positive (trust) and negative (distrust) edges. Many online social services such as Epinions [1] and Slashdot [2] that allow users to express their opinions are naturally represented as signed social graphs. Such graphs have attracted considerable attention [3] for diverse applications including sign prediction [4, 5], link prediction [6–8], node ranking [9–12], community analysis [13–16], graph generation [17, 18], and anomaly detection [19–21]. Node representation learning is a fundamental building block for analyzing graph data, and many researchers have put tremendous efforts into developing effective models for unsigned graphs. Graph convolutional networks (GCN) and their variants [22, 23] have spurred great attention in data mining and machine learning community, and recent works [24, 25] have demonstrated stunning progress by handling the performance degradation caused by over-smoothing [26, 27] (i.e., node representations become indistinguishable as the number of propagation steps increases) or the vanishing gradient problem [25] in the first generation of GCN models. However, all of these models have a limited performance on node representation learning in signed graphs since they only consider unsigned edges under the homophily assumption [22].

Many studies have been recently conducted to consider such signed edges, and they are categorized into network embedding and GCN-based models. Network embedding [28, 29] learns the representations of nodes by optimizing an unsupervised loss that primarily aims to locate two nodes’ embeddings closely (or far) if they are positively (or negatively) connected. However, they are not trained jointly with a specific task in an end-to-end manner, i.e., latent features and the task are trained separately. Thus, their performance is limited unless each of them is tuned delicately. GCN-based models [30, 31] have extended the graph convolutions to signed graphs using balance theory [32] in order to properly propagate node features on signed edges. However, these models are directly extended from existing GCNs without consideration of the over-smoothing problem that degrades their performance (see Fig 4). This problem hinders them from exploiting more information from multi-hop neighbors for learning node features in signed graphs.

**Fig 1:**
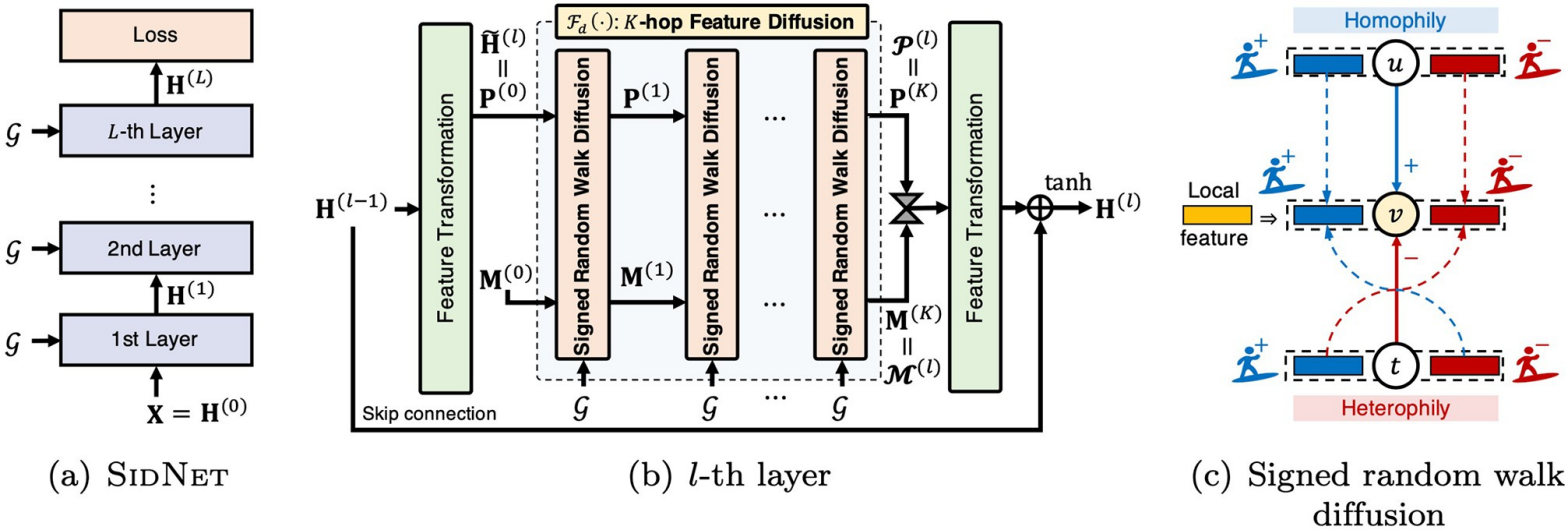
Overall architecture of SidNet. (a) Given a signed graph G and initial node features **X**, SidNet with multiple layers produces the final embeddings **H**^(*L*)^, which is fed to a loss function under an end-to-end framework. (b) A single layer learns node embeddings based on *K*-hop signed random walk diffusions of $F_{d}\left(\cdot \right)$. (c) Our diffusion module aggregates the features of node *v* so that they are similar to those connected by + edges (e.g., node *u*), and different from those connected by − edges (e.g., node *t*). Also, it injects the local feature (i.e., the input feature of each module) of node *v* at each aggregation to make the aggregated features distinguishable.

In this paper, we propose Signed Diffusion Network (SidNet), a novel graph neural network for node representation learning in signed graphs. Our main contributions are summarized as follows:

- **Method.** We propose SidNet, an end-to-end representation learning method in a signed graph with multiple signed diffusion layers (Fig 1). Our signed diffusion layer exploits signed random walks to propagate node embeddings on signed edges, and injects local features (Fig 1). This enables SidNet to learn distinguishable node embeddings effectively considering multi-hop neighbors while preserving local information.
- **Theory.** We theoretically analyze the convergence property (Theorem 1) of our signed diffusion layer, showing how SidNet prevents the over-smoothing issue. We also provide the time complexity analysis (Theorem 2) of SidNet, showing SidNet is linearly scalable w.r.t. the numbers of edges.
- **Experiments.** Extensive experiments show that SidNet effectively learns node representations of signed social graphs for link sign prediction, giving at least 3.3% higher accuracy than the state-of-the-art models in real datasets (Table 3).

The symbols used in this paper are summarized in Table 1. The code of SidNet and datasets are available at https://github.com/snudatalab/SidNet.

**Table 1:** Symbols.

Symbol	Definition
G	signed graph
*n*	numbers of nodes
*m*	numbers of edges
**A**	(*n* × *n*) signed adjacency matrix
**A**_*s*_	(*n* × *n*) adjacency matrix for edges having sign *s*
**D**	(*n* × *n*) diagonal out-degree matrix
*d*_*l*_	dimension of a node embedding at the *l*-th layer
**X**	(*n* × *d*_0_) initial node feature matrix
**H** ^(*l*)^	(*n* × *d*_*l*_) node embedding matrix of the *l*-th layer
*K*	number of diffusion steps
*L*	number of layers
*c*	local injection ratio
$W_{t}^{\left(l\right)}$	(*d*_*l*−1_ × *d*_*l*_) trainable matrix for small feature transformation at the *l*-th layer
**P** ^(*k*)^	(*n* × *d*_*l*_) positive node embedding matrix at the *k*-th diffusion step
**M** ^(*k*)^	(*n* × *d*_*l*_) negative node embedding matrix at the *k*-th diffusion step
$W_{n}^{\left(l\right)}$	(2*d*_*l*_ × *d*_*l*_) trainable matrix that learns a relationship b.t.w. $P^{\left(l\right)}≔{\text{P}}^{\left(K\right)}\&M^{\left(l\right)}≔{\text{M}}^{\left(K\right)}$ at the *l*-th layer
$F_{d}\left(\cdot \right)$	signed random walk diffusion operator of SidNet
;	vertical concatenation of two matrices
||	horizontal concatenation of two matrices
*ϕ*(⋅)	non-linear activator such as tanh

## Related work

### Graph convolutional networks on unsigned graphs

Graph convolutional network (GCN) [22] models the latent representation of a node by employing a convolutional operation on the features of its neighbors. Various GCN-based approaches [22, 23, 33] have aroused considerable attention since they enable diverse graph supervised tasks [22, 34, 35] to be performed concisely under an end-to-end framework. However, the first generation of GCN models exhibit performance degradation due to the over-smoothing and vanishing gradient problems. Several works [26, 27] have theoretically revealed the over-smoothing problem. Also, Li et al. [25] have empirically shown that stacking more GCN layers leads to the vanishing gradient problem as in convolutional neural networks [36]. Consequently, most GCN-based models [22, 23, 33] are shallow; i.e., they do not use the feature information in faraway nodes when modeling node embeddings.

A recent research direction aims at resolving the limitation. Klicpera et al. [24] proposed APPNP exploiting Personalized PageRank [37, 38] to not only propagate hidden node embeddings far but also preserve local features, thereby preventing aggregated features from being over-smoothed. Li et al. [25] suggested ResGCN adding skip connections between GCN layers, as in ResNet [36]. However, all of these models do not provide how to use signed edges since they are based on the homophily assumption [22], i.e., users having connections are likely to be similar, which is not valid for negative edges. As opposed to the homophily, negative edges have the semantics of heterophily [39], i.e., users having connections are dissimilar. Although these methods can still be applied to signed graphs by ignoring the edge signs, their trained features have limited capacity.

### Network embedding and graph convolutional networks on signed graphs

Traditional methods on network embedding extract latent node features specialized for signed graphs in an unsupervised manner. Kim et al. [28] proposed SIDE which optimizes a likelihood over direct and indirect signed connections on truncated random walks sampled from a signed graph. Xu et al. [29] developed SLF considering positive, negative, and non-linked relationships between nodes to learn non-negative node embeddings. However, such approaches are not end-to-end, i.e., they are not directly optimized for solving a supervised task such as link prediction.

There are recent progresses on end-to-end learning on signed networks under the GCN framework. Derr et al. [30] proposed SGCN which extends the GCN mechanism to signed graphs considering balanced and unbalanced relationships supported by structural balance theory [32]. There are several techniques based on attention. Junjie et al. [40] proposed a graph attention network model by incorporating the importance of graph motifs into node feature. Yu et al. [31] reported that their SNEA model outperforms the motif based attention model by combining the graph attention technique and the balanced relationships. However, such state-of-the-art models do not consider the over-smoothing problem since they are directly extended from GCN.

## Proposed method

We propose SidNet (Signed Diffusion Network), a novel end-to-end model for node representation learning in signed graphs. Our SidNet aims to properly aggregate node features on signed edges, and to effectively use the features of multi-hop neighbors so that generated features are not over-smoothed. Our main ideas are to diffuse node features along random walks considering the signs of edges, and to inject local node features at each aggregation.

Fig 1 depicts the overall architecture of SidNet. Given a signed graph G and initial node features $X\in R^{n\times d_{0}}$ as shown in Fig 1, SidNet extracts the final node embeddings $H^{\left(L\right)}\in R^{n\times d_{L}}$ through multiple layers where *n* is the number of nodes, *L* is the number of layers, and *d*_*l*_ is the embedding dimension of the *l*-th layer. Then, **H**^(*L*)^ is fed into a loss function of a specific task so that they are jointly trained in an end-to-end framework. Given **H**^(*l*−1)^, the *l*-th layer aims to learn **H**^(*l*)^ based on feature transformations and signed random walk diffusions of $F_{d}\left(\cdot \right)$ as shown in Fig 1. The layer also uses the skip connection to prevent the vanishing gradient problem when the depth of SidNet increases.

Fig 1 illustrates the intuition behind the signed random walk diffusion. Each node has two features corresponding to positive and negative surfers, respectively. The surfer flips its sign when moving along negative edges, while the sign is kept along positive edges. For example, the positive (or negative) surfer becomes positive at node *v* if it moves from a positively connected node *u* (or a negatively connected node *t*). As a result, the aggregated features at node *v* become similar to those connected by positive edges (e.g., node *u*), and different from those connected by negative edges (e.g., node *t*). In other words, it satisfies homophily and heterophily at the same time while unsigned GCNs cannot handle the heterophily of negative edges. Furthermore, we inject the local feature (i.e., the input feature of the module) of node *v* at each aggregation so that the resulting features remain distinguishable during the diffusion.

### Signed diffusion network

Given a signed graph G and the node embeddings **H**^(*l*−1)^ from the previous layer, the *l*-th layer learns new embeddings **H**^(*l*)^ as shown in Fig 1. It first transforms **H**^(*l*−1)^ into hidden features ${\tilde{H}}^{\left(l\right)}$ as ${\tilde{H}}^{\left(l\right)}=H^{\left(l-1\right)}W_{t}^{\left(l\right)}$ with a learnable parameter $W_{t}^{\left(l\right)}\in R^{d_{l-1}\times d_{l}}$. Then, it applies the signed random walk diffusion which is represented as the function $F_{d}\left(G,{\tilde{H}}^{\left(l\right)}\right)$ which returns $P^{\left(l\right)}\in R^{n\times d_{l}}$ and $M^{\left(l\right)}\in R^{n\times d_{l}}$ as the positive and the negative embeddings, respectively. The embeddings are concatenated and transformed as follows:

$$\begin{array}{c} H^{\left(l\right)}=\phi ([P^{\left(l\right)}\left|\right|M^{\left(l\right)}]W_{n}^{\left(l\right)}+H^{\left(l-1\right)}) \end{array}$$ (1)

where *ϕ*(⋅) is a non-linear activator such as tanh, || denotes horizontal concatenation of two matrices, and $W_{n}^{\left(l\right)}\in R^{2d_{l}\times d_{l}}$ is a trainable weight matrix that learns a relationship between $P^{\left(l\right)}$ and $M^{\left(l\right)}$. We use the skip connection [25, 36] with **H**^(*l*−1)^ in Eq (1) to avoid the vanishing gradient issue which frequently occurs when multiple layers are stacked.

### Signed random walk diffusion

We design the signed random walk diffusion operator $F_{d}\left(\cdot \right)$ used in the *l*-th layer. Given the signed graph G and the hidden node embeddings ${\tilde{H}}^{\left(l\right)}$, the diffusion operator $F_{d}\left(\cdot \right)$ diffuses the node features based on random walks considering edge signs so that it properly aggregates node features on signed edges and prevents the aggregated features from being over-smoothed.

**Fig 2:**
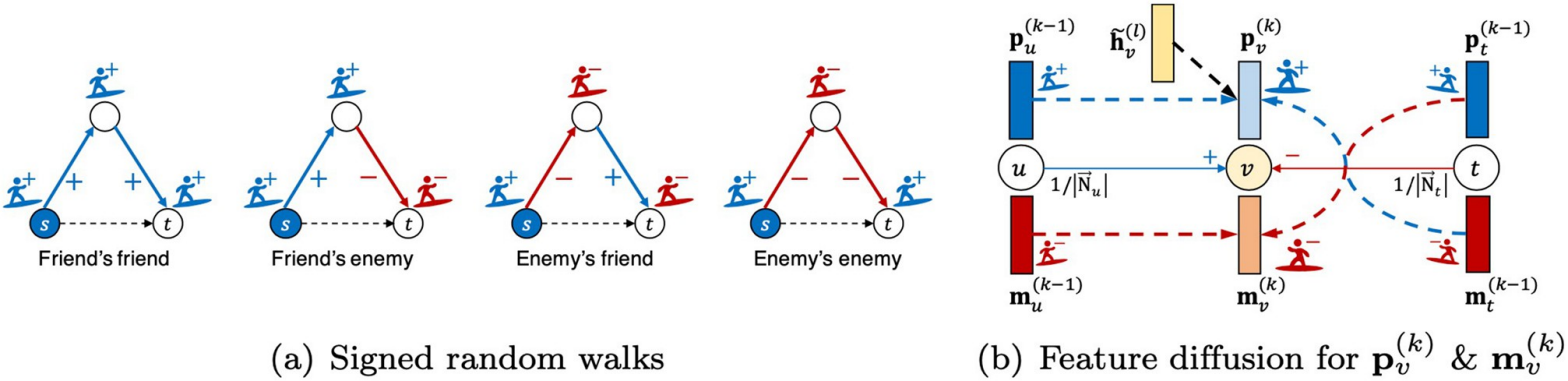
Feature diffusion by signed random walks in SidNet. (a) Signed random walks properly consider edge signs. (b) The positive and the negative feature vectors $p_{v}^{\left(k\right)}$ and $m_{v}^{\left(k\right)}$ are updated from the previous feature vectors and the local feature vector ${\tilde{h}}_{v}^{\left(l\right)}$ as described in Eq (2).

Signed random walks are performed by a signed random surfer [11] who has the + or − sign when moving around the graph. Fig 2 shows signed random walks on four cases according to edge signs: 1) a friend’s friend, 2) a friend’s enemy, 3) an enemy’s friend, and 4) an enemy’s enemy. The surfer starts from node *s* with the + sign. If it encounters a negative edge, the surfer flips its sign from + to −, or vice versa. Otherwise, the sign is kept. The surfer determines whether a target node *t* is a friend of node *s* or not according to its sign.

The diffusion operator $F_{d}\left(\cdot \right)$ exploits the signed random walk for diffusing node features on signed edges. Each node is represented by two feature vectors which represent the positive and negative signs, respectively. Let *k* denote the number of diffusion steps or signed random walk steps. Then, $p_{v}^{\left(k\right)}\in R^{d_{l}\times 1}$ and $m_{v}^{\left(k\right)}\in R^{d_{l}\times 1}$ are aggregated at node *v*, respectively, where $p_{v}^{\left(k\right)}$ (or $m_{v}^{\left(k\right)}$) is the feature vector visited by the positive (or negative) surfer at step *k*. These are recursively obtained by the following equations:

$$\begin{array}{cc} p_{v}^{\left(k\right)} & =\left(1-c\right)(\sum_{u\in {\overset{\leftarrow }{\text{N}}}_{v}^{+}}\frac{p_{u}^{\left(k-1\right)}}{|{\vec{\text{N}}}_{u}|}+\sum_{t\in {\overset{\leftarrow }{\text{N}}}_{v}^{-}}\frac{m_{t}^{\left(k-1\right)}}{|{\vec{\text{N}}}_{t}|})+c{\tilde{h}}_{v}^{\left(l\right)}\\ m_{v}^{\left(k\right)} & =\left(1-c\right)(\sum_{t\in {\overset{\leftarrow }{\text{N}}}_{v}^{-}}\frac{p_{t}^{\left(k-1\right)}}{|{\vec{\text{N}}}_{t}|}+\sum_{u\in {\overset{\leftarrow }{\text{N}}}_{v}^{+}}\frac{m_{u}^{\left(k-1\right)}}{|{\vec{\text{N}}}_{u}|}) \end{array}$$ (2)

where ${\overset{\leftarrow }{\text{N}}}_{v}^{s}$ is the set of incoming neighbors to node *v* connected with edges of sign *s*, ${\vec{\text{N}}}_{u}$ is the set of outgoing neighbors from node *u* regardless of edge signs, ${\tilde{h}}_{v}^{\left(l\right)}$ is the local feature of node *v* (i.e., the *v*-th row vector of ${\tilde{H}}^{\left(l\right)}$), and 0 < *c* < 1 is a local feature injection ratio. That is, the features are computed by the signed random walk feature diffusion with weight 1 − *c* and the local feature injection with weight *c* with the following details. Note that the convergence of Eq (2) is guaranteed as described in Theorem 1; thus, the initial values of $p_{v}^{\left(0\right)}$ and $m_{v}^{\left(0\right)}$ do not affect the final result. In this work, we initialize $p_{v}^{\left(0\right)}$ with ${\tilde{h}}_{v}^{\left(l\right)}$, and randomly initialize $m_{v}^{\left(0\right)}$ in [-1, 1].

#### Signed random walk feature diffusion

Fig 2 illustrates how $p_{v}^{\left(k\right)}$ and $m_{v}^{\left(k\right)}$ are diffused by the signed random walks according to Eq (2). Suppose the positive surfer visits node *v* at step *k*. For this to happen, the positive surfer of an incoming neighbor *u* at step *k* − 1 should choose the edge (*u* → *v*, +) by a probability $1/|{\vec{\text{N}}}_{u}|$. This transition to node *v* along the positive edge allows to keep the surfer’s positive sign. At the same time, the negative surfer of an incoming neighbor *t* at step *k* − 1 should move along the edge (*t* → *v*, −) by a probability $1/|{\vec{\text{N}}}_{t}|$. In this case, the surfer flips its sign from − to +. Considering these signed random walks, $p_{v}^{\left(k\right)}$ is obtained by the weighted aggregation of $p_{u}^{\left(k-1\right)}$ and $m_{t}^{\left(k-1\right)}$. Similarly, $m_{v}^{\left(k\right)}$ is aggregated as shown in Fig 2.

#### Local feature injection

Although the feature diffusion above properly considers edge signs, the generated features could be over-smoothed after many steps if we depend solely on the diffusion. In other words, it considers only the graph information explored by the signed random surfer, while the local information in the hidden feature ${\tilde{h}}_{v}^{\left(l\right)}$ is disregarded during the diffusion. Hence, as shown in Fig 2, we explicitly inject the local feature ${\tilde{h}}_{v}^{\left(l\right)}$ to $p_{v}^{\left(k\right)}$ with weight *c* at each aggregation in Eq (2) so that the diffused features are not over-smoothed. The reason why local features are only injected to + embeddings is that we consider a node should trust (+) its own information (i.e., its local feature).

#### Discussion

Our approach is motivated from SGCN [30], APPNP [24], and SRWR [11, 41]. We describe how we utilize and combine their ideas for developing our method, and how our fusion resolves their limitations when its comes to learning node representation in signed graphs.

- **Motivation from SGCN.** The main idea of SGCN is to make GCN consider balanced and unbalanced paths based on structural balance theory so that the information of balanced paths and that of unbalanced ones are reflected into positive and negative embeddings, respectively. Inspired from this idea, we also maintain two positive and negative embeddings for each node, and make our aggregation phase follow the balance theory. However, simply extending GCN with the balance theory like SGCN does not resolve the over-smoothing issue as shown in Fig 4. Thus, we combine the following ideas of APPNP and SRWR in this framework to overcome the limitation, which are described below.
- **Motivation from APPNP.** To resolve the over-smoothing issue of unsigned GCNs, APPNP utilizes Random Walk with Restart (or personalized pagerank) [42] in a GCN. As a result, APPNP demonstrates that the restart of RWR prevents the over-smoothing problem by inserting input features stochastically during its diffusion (or aggregation) phase. This motivates us to introduce the local feature injection for the same purpose to avoid the issue when learning node embeddings in signed graphs. However, APPNP does not provide a way to deal with signed edges for aggregating node embeddings. To address this challenge, we adopt the signed random walks of SRWR.
- **Motivation from SRWR.** The signed random walks of SRWR were originally proposed for propagating probabilities, not embedding vectors on each node to measure node-to-node similarity scores which are used as a personalized ranking in a signed graph. Thus, this technique had not been studied for learning node representation in signed graphs. Hinted from SGCN and APPNP, we utilize the signed random walks with the local feature injection as shown in Eq (2), and demonstrate that our method effectively considers signed edges while resolving the aforementioned over-smoothing issue.

### Convergence of signed random walk diffusion

Suppose that $P^{\left(k\right)}=\left[p_{1}^{(k)⊤};\cdots ;p_{n}^{(k)⊤}\right]$ and $M^{\left(k\right)}=\left[m_{1}^{(k)⊤};\cdots ;m_{n}^{(k)⊤}\right]$ represent the positive and negative embeddings of all nodes, respectively, where; denotes vertical concatenation. Let **A**_*s*_ be the adjacency matrix for sign *s* such that **A**_*suv*_ is 1 for signed edge (*u* → *v*, *s*), and 0 otherwise. Then, Eq (2) is vectorized as follows:

$$\begin{array}{c} \begin{array}{cc} P^{\left(k\right)} & =\left(1-c\right)\left({\tilde{A}}_{+}^{⊤}P^{\left(k-1\right)}+{\tilde{A}}_{-}^{⊤}M^{\left(k-1\right)}\right)+c{\tilde{H}}^{\left(l\right)}\\ M^{\left(k\right)} & =\left(1-c\right)\left({\tilde{A}}_{-}^{⊤}P^{\left(k-1\right)}+{\tilde{A}}_{+}^{⊤}M^{\left(k-1\right)}\right) \end{array} \end{array}$$ (3)

where ${\tilde{A}}_{s}=D^{-1}A_{s}$ is the normalized matrix for sign *s*, and **D** is a diagonal out-degree matrix (i.e., $D_{ii}=\left|{\vec{\text{N}}}_{i}\right|$). The signed random walk diffusion operator $F_{d}\left(\cdot \right)$ iterates Eq (3) *K* times for 1 ≤ *k* ≤ *K* where *K* is the number of diffusion steps, and it returns $P^{\left(l\right)}\leftarrow P^{\left(K\right)}$ and $M^{\left(l\right)}\leftarrow M^{\left(K\right)}$ as the outputs of the diffusion module at the *l*-th layer.

Furthermore, Eq (3) is compactly represented as

$$\begin{array}{c} \begin{array}{cc} T^{\left(k\right)} & =\left(1-c\right)\tilde{B}T^{\left(k-1\right)}+cQ \end{array} \end{array}$$ (4)

where

$$\begin{array}{c} T^{\left(k\right)}=[\begin{array}{c} P^{\left(k\right)}\\ M^{\left(k\right)} \end{array}]\;\tilde{B}=[\begin{array}{cc} {\tilde{A}}_{+}^{⊤} & {\tilde{A}}_{-}^{⊤}\\ {\tilde{A}}_{-}^{⊤} & {\tilde{A}}_{+}^{⊤} \end{array}]\;Q=[\begin{array}{c} {\tilde{H}}^{\left(l\right)}\\ 0 \end{array}]. \end{array}$$

Then, **T**^(*k*)^ is guaranteed to converge as *k* increases (see Theorem 1).

#### Discussion

According to Eq (5) of Theorem 1, ${\tilde{B}}^{K}\tilde{Q}$ is the node features diffused by *K*-step signed random walks where ${\tilde{B}}^{K}$ is interpreted as the transition matrix of *K*-step signed random walks, and $\tilde{Q}≔cQ$ is the scaled input feature of the diffusion layer. Thus, the approximation is the sum of the diffused features from 1 to *K* steps with a decaying factor 1 − *c*, i.e., the effect of distant nodes gradually decreases while that of neighboring nodes is high. This is the reason why SidNet prevents diffused features from being over-smoothed. Also, the approximation error ∥T* − **T**^(*k*)^∥_1_ exponentially decreases as *K* increases due to the term (1 − *c*)^*K*^. Another point is that the iteration of Eq (3) converges to the same solution no matter what **P**^(0)^ and **M**^(0)^ are given. In this work, we initialize **P**^(0)^ with ${\tilde{H}}^{\left(l\right)}$, and randomly initialize **M**^(0)^ in [−1, 1].

As shown in Fig 1, we use multiple layers in SidNet with non-linear activator tanh(⋅) to increase its learning capacity and model latent non-linear patterns inherent in data. As a result, SidNet performs *K* × *L*-hop feature propagations where *K* and *L* are the numbers of diffusion steps and layers, respectively. One advantage of this approach is that users are able to flexibly control feature propagation and model capacity to suit their own purposes.

### Algorithm of SidNet

Algorithm 1 summarizes SidNet’s overall procedure which is depicted in Fig 1. Given signed adjacency matrix **A** and related hyper-parameters (e.g., numbers *L* and *K* of layers and diffusion steps, respectively), SidNet produces the final hidden node features **H**^(*L*)^ which are fed to a loss function as described in the following section. It first computes the normalized matrices ${\tilde{A}}_{+}$ and ${\tilde{A}}_{-}$ (line 1). Then, it performs the forward function (lines 3 ∼ 12). The forward function repeats the signed random walk diffusion *K* times (lines 6 ∼ 9), and then performs the non-linear feature transformation skip-connected with **H**^(*l*−1)^ (line 11).

**Algorithm 1:** SidNet

**Input:** signed adjacency matrix **A**, initial node feature matrix **X**, number *K* of diffusion steps, number *L* of layers, and local feature injection ratio *c*

**Output:** hidden node feature matrix **H**^(*L*)^

1: compute normalized adjacency matrices for each sign, i.e., ${\tilde{A}}_{+}=D^{-1}A_{+}$ and ${\tilde{A}}_{-}=D^{-1}A_{-}$

2: initialize **H**^(0)^ with **X**

3: **for** *l* ← 1 to *L* **do**    ⊳ *start the forward function of* SidNet

4:  perform the feature transformation as ${\tilde{H}}^{\left(l\right)}\leftarrow H^{\left(l-1\right)}W_{t}^{\left(l\right)}$

5:  initialize **P**^(0)^ with ${\tilde{H}}^{\left(l\right)}$ & randomly initialized **M**^(0)^ ∈ [−1, 1]

6:  **for** *k* ← 1 to *K* **do**    ⊳ *start our SRW diffusion*

7:   $P^{\left(k\right)}\leftarrow \left(1-c\right)\left({\tilde{A}}_{+}^{⊤}P^{\left(k-1\right)}+{\tilde{A}}_{-}^{⊤}M^{\left(k-1\right)}\right)+c{\tilde{H}}^{\left(l\right)}$

8:   $M^{\left(k\right)}\leftarrow \left(1-c\right)\left({\tilde{A}}_{-}^{⊤}P^{\left(k-1\right)}+{\tilde{A}}_{+}^{⊤}M^{\left(k-1\right)}\right)$

9:  **end for**

10:   set $P^{\left(l\right)}\leftarrow P^{\left(K\right)}$ and $M^{\left(l\right)}\leftarrow M^{\left(K\right)}$

11:  compute the *l*-th hidden node features as $H^{\left(l\right)}\leftarrow \text{tanh}\left([P^{\left(l\right)}∥M^{\left(l\right)}\right]W_{n}^{\left(l\right)}+H^{\left(l-1\right)})$

12: **end for**

13: **return H**^(*L*)^

### Loss function for link sign prediction

The link sign prediction task is to predict the missing sign of a given edge. As shown in Fig 1, SidNet produces the final node embeddings **H**^(*L*)^. The embeddings are fed into a loss function $L\left(G,H^{\left(L\right)};\Theta \right)=L_{sign}\left(G,H^{\left(L\right)}\right)+\lambda L_{reg}\left(\Theta \right)$ where **Θ** is the set of model parameters, $L_{sign}\left(\cdot \right)$ is the binary cross entropy loss, and $L_{reg}\left(\cdot \right)$ is the *L*_2_ regularization loss with weight decay *λ*. For a signed edge (*u* → *v*, *s*), the edge feature is $z_{uv}\in R^{2d_{L}\times 1}\;\;=\left[h_{u}^{\left(L\right)};h_{v}^{\left(L\right)}\right]$ where $h_{u}^{\left(L\right)}$ is the *u*-th row vector of **H**^(*L*)^, and; denotes vertical concatenation of two column vectors. Then, $L_{sign}\left(\cdot \right)$ is represented as follows:

$$\begin{array}{c} L_{sign}\left(G,X\right)=-\;\;\;\;\sum_{(u\to v,s)\in E}\;\sum_{t\in \{+,-\}}I\left(t=s\right)\text{log}({\text{softmax}}_{t}(Wz_{uv})) \end{array}$$

where E is the set of signed edges, $W\in R^{2\times 2d_{L}}$ is a learnable weight matrix, softmax_*t*_(⋅) is the probability for sign *t* after softmax operation, and I(·) returns 1 if a given predicate is true, and 0 otherwise.

### Analysis

We first show the convergence guarantee of **T**^(*k*)^, the positive and negative embeddings of all nodes, in Theorem 1 and Lemma 1. Our analysis is inspired from the convergence analysis of [41], which describes the power iteration of a single probability vector on a transition matrix constructed by the signed random walks. In this work, we extend the analysis to the power iteration of multidimensional embedding vectors, and show why our method prevents the over-smoothing issue in Eq (5) (see its interpretation below Eq (4)).

**Theorem 1** *The diffused features in* **T**^(*k*)^ *converge to equilibrium for c* ∈ (0, 1) *as follows*:

$$\begin{array}{cc} T^{*}=\underset{k\to \infty }{\text{lim}}T^{\left(k\right)} & =\underset{k\to \infty }{\text{lim}}(\sum_{i=0}^{k-1}{\left(1-c\right)}^{i}{\tilde{B}}^{i})\tilde{Q}\\ & ={\left(I-\left(1-c\right)\tilde{B}\right)}^{-1}\tilde{Q} \end{array}$$

*where* $\tilde{Q}≔cQ$. *If we iterate* Eq (3) *K times for 1* ≤ *k* ≤ *K*, *the exact solution* T* *is approximated as*

$$\begin{array}{cc} T^{*} & \approx T^{\left(k\right)}\\ & =\tilde{Q}+\left(1-c\right)\tilde{B}\tilde{Q}+\cdots +{\left(1-c\right)}^{K-1}{\tilde{B}}^{K-1}\tilde{Q}+{\left(1-c\right)}^{K}{\tilde{B}}^{K}T^{\left(0\right)} \end{array}$$ (5)

where ∥T* − *T*^(*k*)^∥_1_ ≤ (1 − *c*)^*K*^∥T* − **T**^(0)^∥_1_, *and* = **T**^(0)^ [**P**^(0)^;**M**^(0)^] *is the initial value of* Eq (4).

*proof*. The iteration of Eq (4) is written as follows:

$$\begin{array}{cc} T^{\left(k\right)} & =\left(1-c\right)\tilde{B}T^{\left(k-1\right)}+cQ\\ & ={\left(\left(1-c\right)\tilde{B}\right)}^{2}T^{\left(k-2\right)}+(\left(1-c\right)\tilde{B}+I)\tilde{Q}\\ & =\cdots \\ & ={\left(\left(1-c\right)\tilde{B}\right)}^{k}T^{\left(0\right)}+(\sum_{i=0}^{k-1}({\left(1-c\right)}^{i}{\tilde{B}}^{i}))\tilde{Q}. \end{array}$$ (6)

Note that the spectral radius $\rho (\tilde{B})$ is less than or equal to 1 by Lemma 1; thus, for 0 < *c* < 1, the spectral radius of $\left(1-c\right)\tilde{B}$ is less than 1, i.e., $\rho \left(\left(1-c\right)\tilde{B}\right)=\left(1-c\right)\rho \left(\tilde{B}\right)\leq \left(1-c\right)<1$. Hence, if *k* → ∞, the power of $\left(1-c\right)\tilde{B}$ converges to **0**, i.e., ${\text{lim}}_{k\to \infty }{\left(1-c\right)}^{k}{\tilde{B}}^{k}=0$. Also, the second term in Eq (6) becomes the infinite geometric series of $\left(1-c\right)\tilde{B}$ which converges as the following equation:

$$\begin{array}{c} \begin{array}{cc} T^{*}=\underset{k\to \infty }{\text{lim}}T^{\left(k\right)} & =0+\underset{k\to \infty }{\text{lim}}(\sum_{i=0}^{k-1}({\left(1-c\right)}^{i}{\tilde{B}}^{i}))\tilde{Q}\\ & ={\left(I-\left(1-c\right)\tilde{B}\right)}^{-1}\tilde{Q} \end{array} \end{array}$$

where the convergence always holds if $\rho (\left(1-c\right)\tilde{B})<1$. The converged solution T* satisfies $T^{*}=\left(1-c\right)\tilde{B}T^{*}+cQ$. Also, T* is approximated as Eq (5). Then, the approximation error ∥T* − *T*^(*k*)^∥_1_ is bounded as follows:

$$\begin{array}{c} \begin{array}{cc} {∥T^{*}-T^{\left(k\right)}∥}_{1} & ={∥\left(1-c\right)\tilde{B}T^{*}-\left(1-c\right)\tilde{B}T^{\left(k-1\right)}∥}_{1}\\ & \leq \left(1-c\right)∥\tilde{B}{∥}_{1}{∥T^{*}-T^{\left(k-1\right)}∥}_{1}\\ & \leq \left(1-c\right){∥T^{*}-T^{\left(k-1\right)}∥}_{1}\\ & \leq \cdots \\ & \leq {\left(1-c\right)}^{K}{∥T^{*}-T^{\left(0\right)}∥}_{1} \end{array} \end{array}$$ (7)

where ‖⋅‖_1_ is *L*_1_-norm of a matrix. Note that the bound ${∥\tilde{B}∥}_{1}\leq 1$ of Lemma 1 is used in the above derivation.

**Lemma 1**. *The spectral radius of* $\tilde{B}$ *in* Eq (3) *is less than or equal to* 1, *i.e*., $\rho \left(\tilde{B}\right)\leq {∥\tilde{B}∥}_{1}\leq 1$.

*Proof*. According to spectral radius theorem [43], $\rho \left(\tilde{B}\right)\leq {∥\tilde{B}∥}_{1}$ where ‖⋅‖_1_ denotes *L*_1_-norm of a given matrix, indicating the maximum absolute column sum of the matrix. Note that the entries of $\tilde{B}$ are non-negative probabilities; thus, the absolute column sums of $\tilde{B}$ are equal to its column sums which are obtained as follows:

$$\begin{array}{c} \begin{array}{cc} 1_{2n}^{⊤}\tilde{B} & =[\begin{array}{cc} 1_{n}^{⊤}{\tilde{A}}_{+}^{⊤}+1_{n}^{⊤}{\tilde{A}}_{-}^{⊤} & 1_{n}^{⊤}{\tilde{A}}_{-}^{⊤}+1_{n}^{⊤}{\tilde{A}}_{+}^{⊤} \end{array}]\\ & =[\begin{array}{cc} 1_{n}^{⊤}{\tilde{A}}^{⊤} & 1_{n}^{⊤}{\tilde{A}}^{⊤} \end{array}]\\ & =[\begin{array}{cc} b^{⊤} & b^{⊤} \end{array}] \end{array} \end{array}$$

where ${\tilde{A}}^{⊤}={\tilde{A}}_{+}^{⊤}+{\tilde{A}}_{-}^{⊤}$, and **1**_*n*_ is an *n*-dimensional one vector. Note that ${\tilde{A}}_{s}^{⊤}=A_{s}^{⊤}D^{-1}$ for sign *s* where **D** is a diagonal out-degree matrix (i.e., $D_{uu}=\left|{\vec{\text{N}}}_{u}\right|$). Then, $1_{n}^{⊤}{\tilde{A}}^{⊤}$ is represented as

$$\begin{array}{c} 1_{n}^{⊤}{\tilde{A}}^{⊤}=1_{n}^{⊤}\left(A_{+}^{⊤}+A_{-}^{⊤}\right)D^{-1}=1_{n}^{⊤}{\left|A\right|}^{⊤}D^{-1}=\left(|A\right|1_{n}{)}^{⊤}D^{-1}=b^{⊤} \end{array}$$

where **|** **A** **|** = **A**_+_+ **A**_−_ is the absolute adjacency matrix. The *u*-th entry of |**A**|**1**_*n*_ indicates the out-degree of node *u*, denoted by $|{\vec{\text{N}}}_{u}|$. Note that $D_{uu}^{-1}$ is $1/|{\vec{\text{N}}}_{u}|$ if *u* is a non-deadend. Otherwise, $D_{uu}^{-1}=0$ (i.e., a deadend node has no outgoing edges). Hence, the *u*-th entry of **b**^⊤^ is 1 if node *u* is not a deadend, or 0 otherwise; its maximum value is less than or equal to 1. Therefore, $\rho \left(\tilde{B}\right)\leq {∥\tilde{B}∥}_{1}\leq 1$.

#### Complexity analysis

We analyze the time complexity of SidNet as follows.

**Theorem 2** (Time Complexity of SidNet). *The time complexity of the l-th layer is O*(*Kmd*_*l*_+ *nd*_*l*−1_ *d*_*l*_) *where K is the number of diffusion steps*, *d*_*l*_ *is the feature dimension of the l*-*th layer, and m and n are the number of edges and nodes, respectively. Assuming all of d*_*l*_ *are set to d*, SidNet *with L layers takes O*(*LKmd* + *Lnd*^2^) *time*.

*Proof*. The feature transform operations require *O*(*nd*_*l*−1_ *d*_*l*_) time due to their dense matrix multiplication. Each iteration of the signed random walk diffusion in Eq (3) takes *O*(*md*_*l*_) time due to the sparse matrix multiplication $\tilde{B}T^{\left(k-1\right)}$ where the number of non-zeros of $\tilde{B}$ is *O*(*m*). Thus, *O*(*Kmd*_*l*_) is required for *K* iterations. Overall, the total time complexity of the *l*-th layer is *O*(*Kmd*_*l*_+ *nd*_*l*−1_ *d*_*l*_).

Theorem 2 indicates that given the hyperparameters, SidNet exhibits the linear scalability w.r.t. the number *m* of edges.

## Experiments

We evaluate the effectiveness of SidNet through the link sign prediction task on real-world signed graphs. Specifically, we aim to answer the following questions:

- **Q1. Link sign prediction.** How effective is our proposed SidNet for predicting the signs of missing edges compared to state-of-the-art methods?
- **Q2. Ablation study.** How does each component of SidNet affect node representation learning in connection with the link sign prediction?
- **Q3. Effect of local injection ratio.** How does the ratio *c* of the local feature injection in SidNet affect the performance of link sign prediction?
- **Q4. Effect of propagation hops.** How does propagation hops of SidNet affect the performance of the link sign prediction?
- **Q5. Effect of embedding dimension.** How does the dimension of embeddings produced by SidNet affect the accuracy of link sign prediction compared to other methods?

### Experimental setting

#### Datasets

We perform experiments on five signed graphs summarized in Table 2. The Bitcoin-Alpha and Bitcoin-OTC datasets [5] are extracted from directed online trust networks served by Bitcoin Alpha and Bitcoin OTC, respectively. The Wikipedia dataset [44] is a signed graph representing the administrator election procedure in Wikipedia where a user can vote for (+) or against (−) a candidate. The Slashdot dataset [2] is collected from Slashdot, a technology news site which allows a user to create positive or negative links to others. The Epinions dataset [1] is a directed signed graph scraped from Epinions, a product review site in which users mark their trust or distrust to others.

**Table 2:** Dataset statistics of directed signed graphs. |V| and |E| are the number of nodes and edges, respectively. Given sign *s* ∈ {+, −}, |E^*s*^| and *ρ*(*s*) are the number and percentage of edges with sign *s*, respectively. The local injection ratio is denoted by *c*.

Dataset	|V|	|E|	|E^+^|	|E^−^|	*ρ*(+)	*ρ*(−)	*c*
Bitcoin-Alpha^1^	3,783	24,186	22,650	1,536	94%	6%	0.35
Bitcoin-OTC^2^	5,881	35,592	32,029	3,563	90%	10%	0.25
Wikipedia^3^	7,118	103,675	81,318	22,357	78%	22%	0.45
Slashdot^4^	79,120	515,397	392,326	123,071	76%	24%	0.55
Epinions^5^	131,828	841,372	717,667	123,705	85%	15%	0.55

^1^ https://snap.stanford.edu/data/soc-sign-bitcoin-alpha.html

^2^ https://snap.stanford.edu/data/soc-sign-bitcoin-otc.html

^3^ https://snap.stanford.edu/data/wiki-Elec.html

^4^ http://konect.cc/networks/slashdot-zoo

^5^ http://www.trustlet.org/extended_epinions.html

The publicly available signed graphs do not contain initial node features even though they have been utilized as representative datasets in signed graph analysis. Due to this reason, many previous works [30, 31] on GCN for signed graphs have exploited singular vector decomposition (SVD) to extract initial node features. Thus, we follow their setup, i.e., **X** = **U** **Σ**_*d*_ is the initial feature matrix for all GCN-based models where $A≃U\Sigma_{d_{i}}V^{⊤}$ is obtained by a truncated SVD method, called Randomized SVD [45], with target rank *d*_*i*_ = 128. Note that the method is very efficient (i.e., its time complexity is $O(nd_{i}^{2})$ where *n* is the number of nodes) and performed only once as a preprocessing in advance; thus, it has little effect on the computational performance of training and inference.

#### Competitors

We compare our proposed SidNet with the following competitors:

- **SRWR**[11, 41] is a personalized ranking method for measuring trustworthiness scores between nodes based on signed random walks. In [41], they used the Wikipedia, Slashdot, and Epinions datasets as directed graphs without preprocessing. They randomly selected 2, 000 seed nodes and choose 20% edges of positive and negative links of each node as validation and test sets. The remaining edges are used as a training set. They measured accuracy (i.e., the ratio of correct predictions) and macro F1 score for the task.
- **APPNP** [24] is an unsigned GCN model based on Personalized PageRank.
- **ResGCN** [25] is another unsigned GCN model exploiting skip connections to stack multiple layers.
- **SIDE** [28] is a network embedding model optimizing the likelihood over signed edges using random walk sequences to encode structural information into node embeddings. In [28], they used the Wikipedia, Slashdot, and Epinions datasets as directed graphs without preprocessing, and performed 5-fold cross validation. They measured AUC and F1 score for the task.
- **SLF** [29] is another network embedding model considering positive, negative, and non-linked relationships to learn non-negative node embeddings. In [29], they used the Wikipedia, Slashdot, and Epinions datasets as directed graphs without preprocessing. They randomly split each dataset into training and test sets by the 8:2 ratio. They used AUC and F1 score for the task.
- **SGCN** [30] is a state-of-the-art signed GCN model considering balanced and unbalanced paths motivated from balance theory to propagate embeddings. In [30], they used the Bitcoin-Alpha, Bitcoin-OTC, Slashdot, and Epinions datasets. They modified each dataset so that the resulting graph becomes undirected, and filtered out nodes with few links randomly from the two larger networks (Slashdot and Epinions). For each graph, they randomly split edges into training and test sets by the 8:2 ratio. They used AUC and F1 score for the task.
- **SNEA** [31] is another signed GCN model extending SGCN by learning attentions on the balanced and unbalanced paths for modeling embeddings. According to [31], the experimental setup of SNEA is the same as that of SGCN.

Note that each dataset originally represents a directed graph, not an undirected graph. Thus, we test all methods including SGCN and SNEA in directed graphs formed from non-filtered original datasets. Also, APPNP and ResGCN are originally designed for unsigned graphs (i.e., they were not tested for the sign prediction task in [24, 25]). In this work, we use the absolute adjacency matrix for APPNP and ResGCN.

#### Implementation and machines

All methods are implemented by PyTorch and Numpy in Python. We use a machine with Intel E5-2630 v4 2.2GHz CPU and Geforce GTX 1080 Ti.

#### Data split and evaluation metrics

We randomly split the edges of a signed graph into training and test sets by the 8:2 ratio. As shown in Table 2, the sign ratio is highly skewed to the positive sign, i.e., the sampled datasets are naturally imbalanced. Considering the class imbalance, we measure the area under the curve (AUC) to evaluate predictive performance. We also report macro F1 measuring the average of the ratios of correct predictions for each sign since negative edges need to be treated as important as positive edges (i.e., it gives equal importance to each class). A higher value of AUC or macro F1 indicates better performance. We repeat each experiment 10 times with different random seeds and report the average and standard deviation of test values.

#### Hyperparameter settings

We set the dimension of final node embeddings to 32 for all methods so that their embeddings have the same learning capacity for the target task. We perform 5-fold cross-validation for each method to find the best hyperparameters and measure the test accuracy with the selected ones. In the cross-validation for SidNet, the local injection ratio *c* is selected from 0.05 to 0.95 by step size 0.1. We set the number *L* of layers to 2, the number *K* of diffusion steps to 10, and the feature dimension *d*_*l*_ of each layer to 32. We follow the range of each hyperparameter recommended in its corresponding paper for the cross-validation of other models. Our model is trained by the Adam optimizer [46], where the learning rate is 0.01, the weight decay *λ* is 0.001, and the number of epochs is 100.

### Link sign prediction

We evaluate the performance of each method on link sign prediction. Tables 3 and 4 summarize the experimental results in terms of AUC and macro F1, respectively. Note that our SidNet shows the best performance in terms of AUC and macro F1 scores. SidNet presents 3.3 ∼ 6.6% and 1.6 ∼ 7.4% improvements over the second best models in terms of AUC and macro F1, respectively. We have the following observations.

- SidNet outperforms an unsupervised method SRWR for the link sign prediction over all datasets; this implies learning node embedding with the signed random walks and the local feature injection is more effective for the task.
- The unsigned GCN models APPNP and ResGCN show worse performance than SidNet, which shows the importance of using sign information.
- The performance of network embedding techniques such as SIDE and SLF is worse than that of other GCN-based models; this shows the importance of jointly learning for feature extraction and link sign prediction for the performance.
- The performance of SGCN and SNEA which use limited features from nodes within 2 ∼ 3 hops is worse than that of SidNet which exploits up to *K* × *L*-hop neighbors’ features for each layer where *K* is set to 10, and *L* is set to 2 in these experiments. It indicates that carefully exploiting features from distant nodes as well as neighboring ones is crucial for the performance.

**Table 3:** SidNet gives the best link sign prediction performance in terms of AUC. The best model is in bold, and the second best model is underlined. The % increase measures the best accuracy against the second best accuracy.

AUC	Bitcoin-Alpha	Bitcoin-OTC	Wikipedia	Slashdot	Epinions
**SRWR**	0.808±0.011	0.859±0.010	0.762±0.004	0.754±0.002	0.907±0.001
**APPNP**	0.854±0.010	0.867±0.009	0.756±0.034	0.837±0.003	0.870±0.002
**ResGCN**	0.853±0.017	0.876±0.010	0.816±0.018	0.744±0.004	0.871±0.002
**SIDE**	0.801±0.020	0.839±0.013	0.736±0.026	0.814±0.003	0.880±0.003
**SLF**	0.779±0.023	0.797±0.014	0.869±0.021	0.833±0.006	0.876±0.005
**SGCN**	0.824±0.018	0.857±0.008	0.768±0.015	0.827±0.004	0.895±0.002
**SNEA**	0.855±0.006	0.858±0.008	0.764±0.009	0.754±0.005	0.771±0.004
**SidNet** **(proposed)**	**0.908±0.005**	**0.920±0.004**	**0.910±0.002**	**0.892±0.001**	**0.937±0.002**
**% increase**	6.1%	4.7%	4.7%	6.6%	3.3%

**Table 4:** SidNet gives the best link sign prediction performance in terms of macro F1. The best model is in bold, and the second best model is underlined. The % increase measures the best accuracy against the second best accuracy.

macro F1	Bitcoin-Alpha	Bitcoin-OTC	Wikipedia	Slashdot	Epinions
**SRWR**	0.687±0.010	0.740±0.007	0.706±0.004	0.669±0.002	0.776±0.001
**APPNP**	0.682±0.005	0.762±0.009	0.636±0.013	0.748±0.003	0.773±0.004
**ResGCN**	0.658±0.006	0.735±0.015	0.677±0.006	0.609±0.004	0.784±0.003
**SIDE**	0.663±0.008	0.709±0.008	0.632±0.008	0.685±0.009	0.785±0.006
**SLF**	0.615±0.027	0.641±0.025	0.761±0.028	0.733±0.008	0.810±0.008
**SGCN**	0.690±0.014	0.776±0.008	0.624±0.012	0.752±0.013	0.844±0.002
**SNEA**	0.670±0.005	0.742±0.011	0.702±0.007	0.690±0.005	0.805±0.005
**SidNet** **(proposed)**	**0.757±0.012**	**0.799±0.007**	**0.792±0.005**	**0.782±0.002**	**0.857±0.001**
**% increase**	7.4%	1.6%	4.1%	4.0%	1.6%

### Ablation study

We examine the effectiveness of each component used in SidNet through an ablation study. As a baseline, we consider the signed random walk diffusion (SRWDiff) of a single layer with no other components, which is achieved by setting *c* = 0, *K* = 10, and *L* = 1. Then, we combine SRWDiff with the local feature injection (LFI) by setting *c* > 0 where the value of *c* varies with datasets. As seen in the second row of Table 5, this combination significantly improves AUC of the link sign prediction, especially in Wikipedia and Slashdot datasets. This emphasizes the importance of injecting local features into the signed random walk diffusion process. Further, the performance slightly increases by using multiple layers (ML) with the skip connection (SC) over all datasets as shown in the fourth row of Table 5.

**Table 5:** Ablation study results on SidNet in terms of AUC. The accuracy considerably improves by combining the signed random walk diffusion (SRWDiff) and the local feature injection (LFI). Using multiple layers (ML) together with the skip connection (SC) leads to the best performance of SidNet across all tested datasets.

Methods	*c*	*K*	*L*	Bitcoin-Alpha	Bitcoin-OTC	Wikipedia	Slashdot	Epinions
**SRWDiff**	0	10	1	0.817±0.015	0.836±0.007	0.673±0.005	0.658±0.005	0.823±0.003
**SRWDiff+LFI**	>0	10	1	0.908±0.006	0.917±0.004	0.904±0.002	0.888±0.001	0.934±0.001
**SRWDiff+LFI+ML**	>0	10	2	0.903±0.014	0.918±0.003	0.906±0.003	0.888±0.003	0.934±0.003
**SRWDiff+LFI+ML+SC (final)**	>0	10	2	**0.908±0.005**	**0.920±0.004**	**0.910±0.002**	**0.892±0.001**	**0.937±0.002**

### Effect of local injection ratio

**Fig 3:**
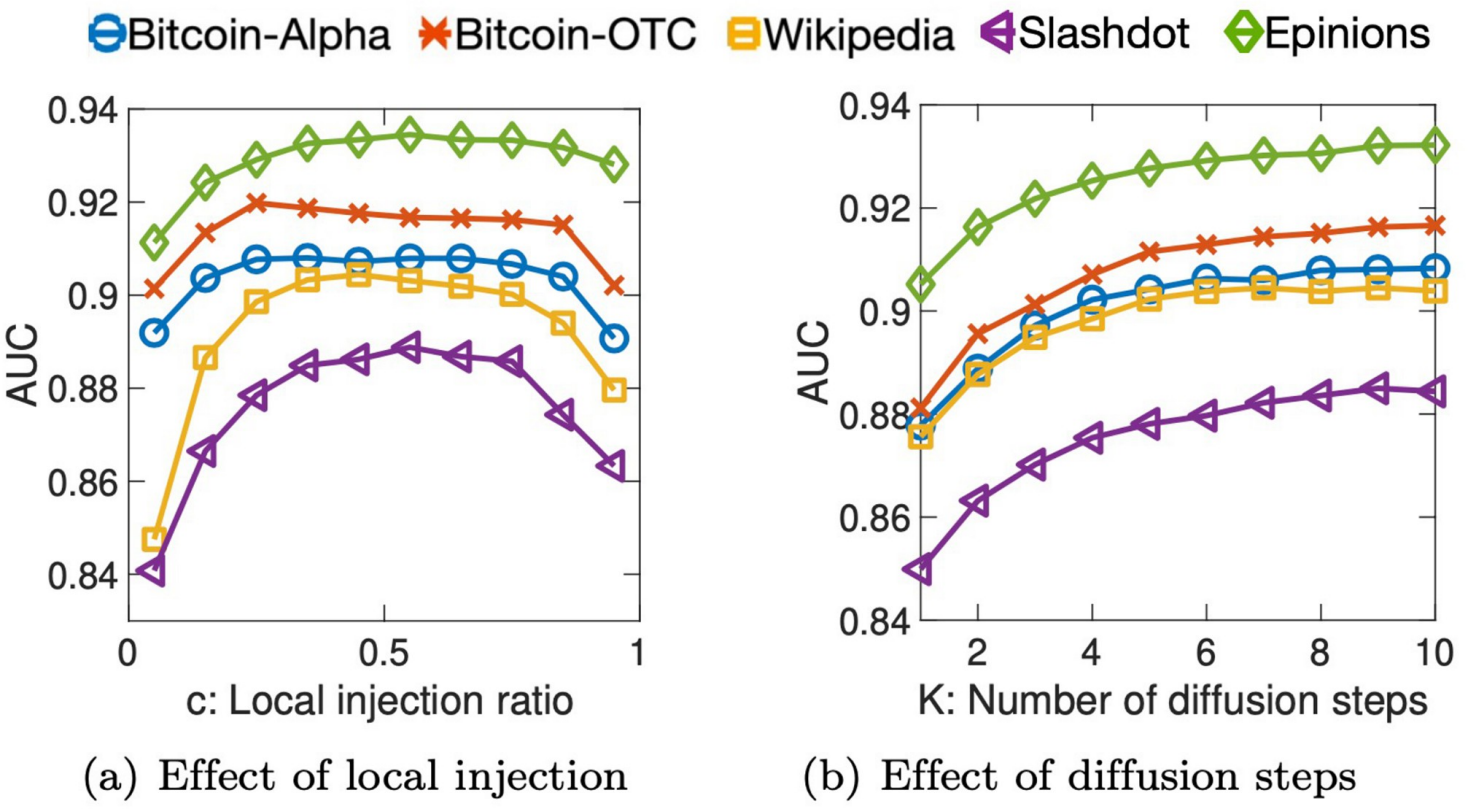
Effects of the local injection ratio *c* and the number *K* of diffusion steps of SidNet. (a) A relatively small value (0.15∼0.35) of *c* is the best for Bitcoin-Alpha and Bitcoin-OTC while *c* around 0.5 shows the best accuracy for the others. (b) The performance of SidNet improves and converges as *K* increases (Theorem 1).

We examine the effect of the local injection ratio *c* in the diffusion module of SidNet. We use one layer, and set the number *K* of diffusion steps to 10; we vary *c* from 0.05 to 0.95 by 0.1, and measure the performance of the link sign prediction task in terms of macro F1. Fig 3 shows the effect of *c* for the predictive performance of SidNet. For small datasets such as Bitcoin-Alpha and Bitcoin-OTC, *c* between 0.15 and 0.35 provides the best performance. On the other hand, *c* around 0.5 shows the best accuracy for Wikipedia, Slashdot, and Epinions datasets. For all datasets, a too low or too high value of *c* (e.g., 0.05 or 0.95) results in a poor performance. For each dataset, we select the value of *c* producing the best accuracy in Fig 3, and record it in Table 2 for the following experiments.

### Effect of propagation hops

We investigate the effect of the propagation hop count with which features are propagated in SidNet for learning from signed graphs. As described in Theorem 1, the hop count of SidNet is determined by *K* × *L* where *K* and *L* are the numbers of diffusion steps and layers, respectively. Thus, we examine the effects of either or both of *K* and *L*. In these experiments, we use the local injection ratio *c* in Table 2 for each dataset.

#### Effect of the number *K* of diffusion steps

To see its pure effect, we use one layer (*L* = 1) so that the hop count is decided by only the number *K* of diffusion steps. We vary *K* from 1 to 10 and evaluate the performance of SidNet in terms of AUC for each diffusion step. Fig 3 shows that the performance of SidNet gradually improves over all datasets as the hop count increases. Note that the performance of SidNet converges in general after a sufficient number of diffusion steps, which is from Theorem 1.

#### Effect of the number *L* of layers

**Fig 4:**
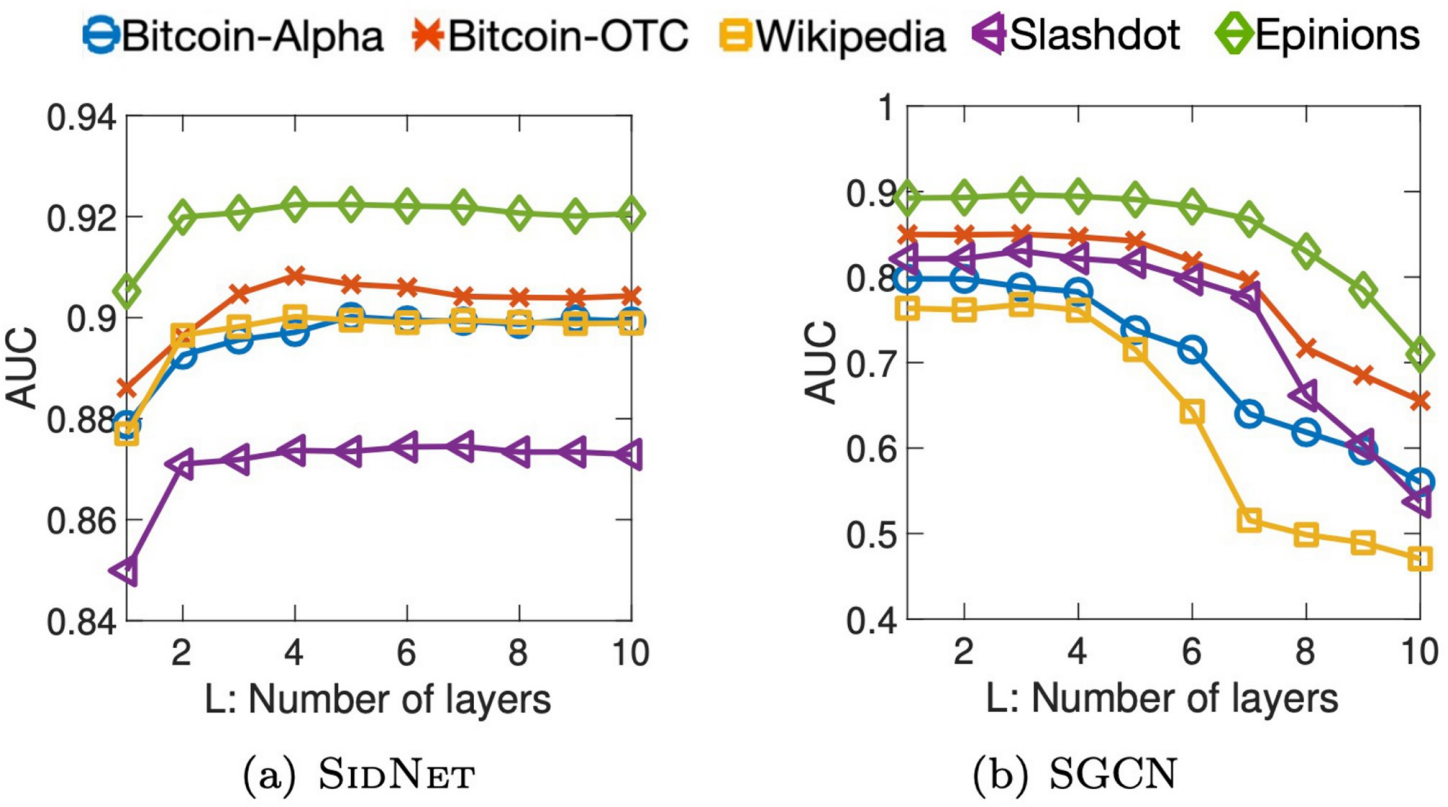
Effect of the number *L* of layers of SidNet compared to the state-of-the-art SGCN. The accuracy of SidNet increases and becomes stable while that of SGCN dramatically degrades as *L* increases.

In this experiment, we set *K* to 1 so that the hop count is decided by only the number *L* of layers. We increase *L* from 1 to 10, and compare SidNet to SGCN, the state-of-the-art-model for learning from signed graphs. The hop count of SGCN is also determined by its number of layers. Fig 4 shows that the performance of SidNet gradually improves as *L* increases while that of SGCN dramatically decreases over all datasets. This indicates that SGCN suffers from the performance degradation problem when its network becomes deep, i.e., it is difficult to use information beyond 3-hop neighbors in SGCN. On the other hand, SidNet utilizes features of farther nodes, and generates more expressive and stable embedding than SGCN does.

#### Effect of both *K* and *L*

**Fig 5:**
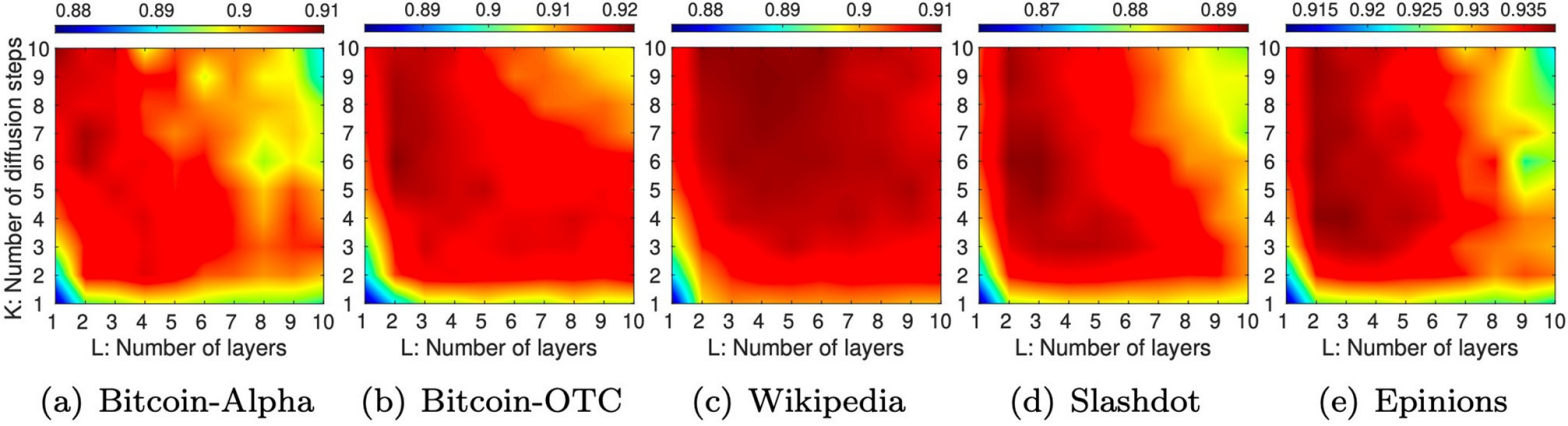
Effect of propagation hops of SidNet in terms of AUC where SidNetperforms *K* × *L*-hop feature propagations. Overall, the accuracy becomes better by setting *K* and *L* such that 20 ≤ *K* × *L* (hop count) ≤ 30 while SidNet with a small hop count exhibits poor results over all datasets.

We further vary both *K* and *L* to investigate the effect of hop counts which are determined by *K* × *L* where 1 ≤ *K*, *L* ≤ 10. Fig 5 demonstrates the AUC’s tendency in the link sign prediction, with the following observations:

- SidNet produces a better accuracy when the hop count is between 20 and 30 in general. On the other hand, a small hop count results in inferior performance over all tested datasets.
- Overall, the upper left triangle of each plot are redder than the lower right triangle, implying *K* of our diffusion module (or diffusing features via signed random walks) is more influential in the performance of SidNet than *L* (or simply stacking layers).

### Effect of embedding dimension

**Fig 6:**
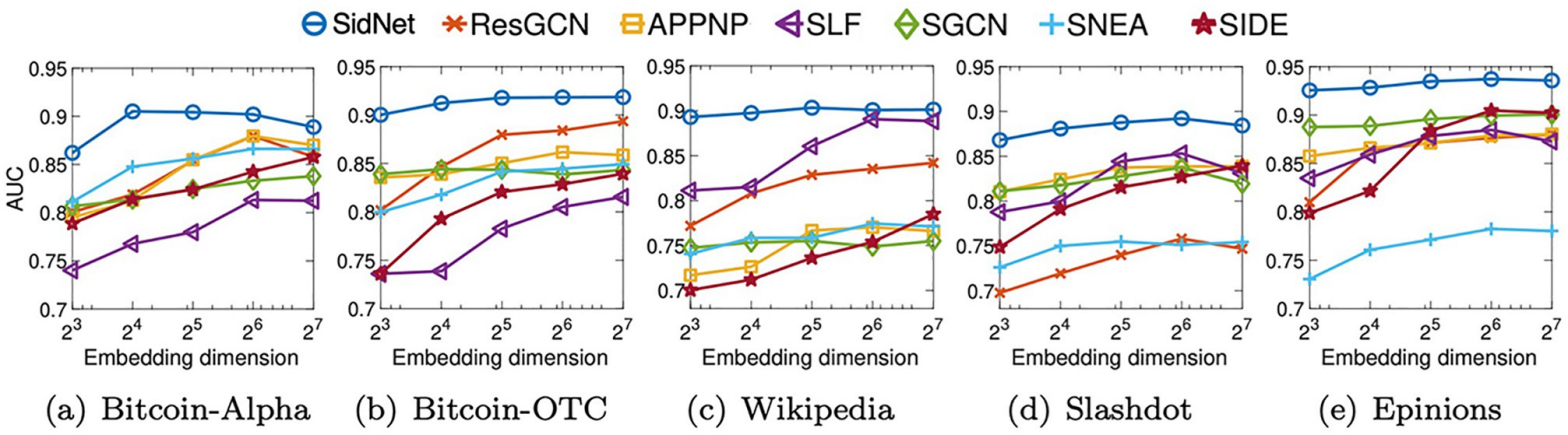
Effect of embedding dimension of each model. SidNet gives a stable performance over varying embedding dimensions, and outperforms other state-of-the-art methods.

We investigate the effect of the node embedding dimension of each model for the link sign prediction task. For this experiment, we vary the dimension of hidden and final node embeddings from 8 to 128 where other hyperparameters of each model are set to those producing the results in Table 3. Then, we observe the trend of AUC in the link sign prediction task. As shown in Fig 6, SidNet significantly outperforms its competitors over all the tested dimensions, and it is relatively less sensitive to the embedding dimension than other models in all datasets except the Bitcoin-Alpha dataset.

## Conclusion

In this paper, we propose Signed Diffusion Network (SidNet), a novel graph neural network that performs end-to-end node representation learning for link sign prediction in signed graphs. We propose a signed random walk diffusion method to properly diffuse node features on signed edges, and suggest a local feature injection method to make diffused features distinguishable. Our diffusion method empowers SidNet to effectively train node embeddings considering multi-hop neighbors while preserving local information. Our extensive experiments show that SidNet provides the best accuracy outperforming the state-of-the-art models in link sign prediction. Future research directions include analyzing our model for graph reconstruction and clustering in signed graphs, and extending it for multi-view networks.
